# Human fascioliasis in Africa: A systematic review

**DOI:** 10.1371/journal.pone.0261166

**Published:** 2021-12-09

**Authors:** Veronique Dermauw, Joan Muchai, Yara Al Kappany, Ana Lucia Fajardo Castaneda, Pierre Dorny

**Affiliations:** 1 Department of Biomedical Sciences, Institute of Tropical Medicine, Antwerp, Belgium; 2 Somalia Country Office, Food and Agriculture Organisation of the United Nations (FAO), Nairobi, Kenya; 3 Department of Parasitology, Faculty of Veterinary Medicine, Mansoura University, Mansoura, Egypt; 4 Department of Virology, Parasitology and Immunology, Faculty of Veterinary Medicine, Ghent University, Merelbeke, Belgium; Dokkyo Medical University, JAPAN

## Abstract

Fascioliasis is a globally distributed, parasitic zoonosis, caused by *Fasciola hepatica* and *F*. *gigantica*. A comprehensive overview of the epidemiology of human fascioliasis in Africa is missing up to now. Therefore, our objective was to conduct a systematic review aiming to summarize recent knowledge on the distribution, prevalence, and risk factors of human fascioliasis in Africa. A key word search was performed in PubMed, Web of Science and Africa Wide, to gather relevant literature, published between the 1st of January 2000 and 31st of December 2020. A total of 472 records were initially retrieved, with 40 full text articles retained for the qualitative synthesis. Human fascioliasis was reported in 12 African countries, namely Algeria, Angola, Cape Verde, Egypt, Ethiopia, Ghana, Morocco, Nigeria, Senegal, South-Africa, Tanzania and Tunisia. The majority of the studies was conducted in Egypt. A total of 28 records were population surveys. Coproscopy was the most commonly used tool for fascioliasis diagnosis in these surveys. Gender (being female), consumption of raw vegetables/seeds, age, owning livestock, and use of unsafe drinking water sources, were identified as risk factors in 7 studies. Furthermore, 43 case reports were retrieved, described in 12 studies. Eosinophilia was present in 39 of these cases, while 11 had positive coproscopy results. Eight cases described having eaten raw wild vegetables. Overall, the low number and quality of records retrieved indicates that human fascioliasis remains a truly neglected disease in Africa, and more epidemiological studies are urgently needed to both establish the actual distribution as well as risk factors on the continent.

## Introduction

Fascioliasis is a globally distributed, parasitic zoonosis, caused by the liver flukes, *Fasciola hepatica* and *F*. *gigantica*. These parasites have a complex life cycle involving an intermediate snail host, a carrier (i.e. aquatic plants) and a final mammal host (e.g. cattle, sheep but also humans). Livestock acquire the infection when grazing on contaminated pastures, while humans typically become infected through consumption of raw water plants (e.g., watercress or others) contaminated with encysted metacercariae of *Fasciola* spp. [1]. Other infection modes such as transmission via contaminated water or cooking utensils have been suggested as well [1].

In livestock, for decades fascioliasis has been a well-known disease, with a significant economic impact in the agricultural sector, due to liver condemnation, poor carcass quality, and reduced growth rate and milk production in ruminants. Global estimates of these financial losses are still lacking, yet in South-East Asia, these were estimated to range between AU$4 billion and AU$11 billion annually [2]. Due to its importance in the agricultural sector, researchers have intensively studied factors favouring transmission from the snail to animal host, such as e.g. rainfall and temperature [3]. Moreover, a mathematical transmission model was developed, describing fascioliasis infection dynamics in herds, and allowing the evaluation of control strategies [4].

In contrast, human fascioliasis has historically been considered of secondary importance, the disease only started to receive some attention from the 1990s onwards [5, 6]. Globally, 2.6 million people are estimated to be infected with *Fasciola* spp., and over 180 million are thought to be at risk [1, 7]. Globally, the disease is estimated to incur 90,000 Disability Adjusted Life Years (DALYs) [7], due to the associated abdominal problems. This estimate, however, does not yet account for the immunosuppression, neurological or ocular effects due to fascioliasis, the actual burden could thus even be higher [6, 8]. Human fascioliasis is an emerging disease [6, 9, 10], and a further increase in incidence might be expected due to global warming influencing intermediate host abundance and parasite transmission [11]. In response, fascioliasis was listed by WHO as a neglected tropical disease (NTD) in 2010 [12].

In Africa, there’s an overlapping distribution of *F*. *hepatica* and *F*. *gigantica*. The presence of *F*. *hepatica* is mainly restricted to the Mediterranean area (i.e., Maghreb countries, such as Morocco, Algeria and Tunisia, as well as Egypt and Libya), southern Zimbabwe, South Africa, Lesotho, as well as to some areas at higher altitude in Kenya, Tanzania and Ethiopia, while *F*. *gigantica* is present throughout most of the continent [13, 14]. Animal fascioliasis has been reported in 13 countries (i.e. Botswana, Chad, Egypt, Ethiopia, Kenya, Nigeria, South Africa, Sudan, Tanzania, Tunisia, Uganda, Zambia and Zimbabwe) (between 2000 and 2015), with prevalence estimates in ruminants up to 91% [15, 16].

In contrast, important knowledge gaps persist with regard to the occurrence of human fascioliasis in Africa. In Egypt, human fascioliasis is an emerging disease [13, 17, 18], exhibiting a seasonal pattern, with a peak of infections being observed in August [17]. Consumption of wild vegetables, and terrestrial cultivated plants, irrigated and washed prior to consumption, as well as use of contaminated drinking water have been listed as risk factors for the disease in Egypt [19]. The occurrence of human fascioliasis in other African countries is however less clear. The WHO has listed 15 countries on the continent as having reported cases of the disease [20], although it is not clear on what basis this was done. Esteban et al. (1998) [13] on the other hand, retrieved published case reports for Algeria, Egypt, Morocco, Tunisia and Zimbabwe only. Up to now, a comprehensive overview of current knowledge on human fascioliasis epidemiology in Africa is missing. Our aim, therefore, was to review recent literature to summarize the distribution, prevalence and risk factors of human fascioliasis in Africa.

## Materials and methods

### Information sources and search strategy

A systematic review was conducted aiming to map the body of literature on human fascioliasis in Africa, published between the 1st of January 2000 and 31st of December 2020 (S1 File). Three scientific databases, namely PubMed, Web of Science and CAB Direct were searched, using a search phrase that combined search terms about humans, fascioliasis and Africa, the latter part based on the search phrase developed by Pienaar et al. [21] (for the full search phrase, and translated search phrase for each of the databases: S1 File). Moreover, reference lists of review articles were screened for relevant records, these were added as additional records.

### Study selection and eligibility criteria

**Fig 1:**
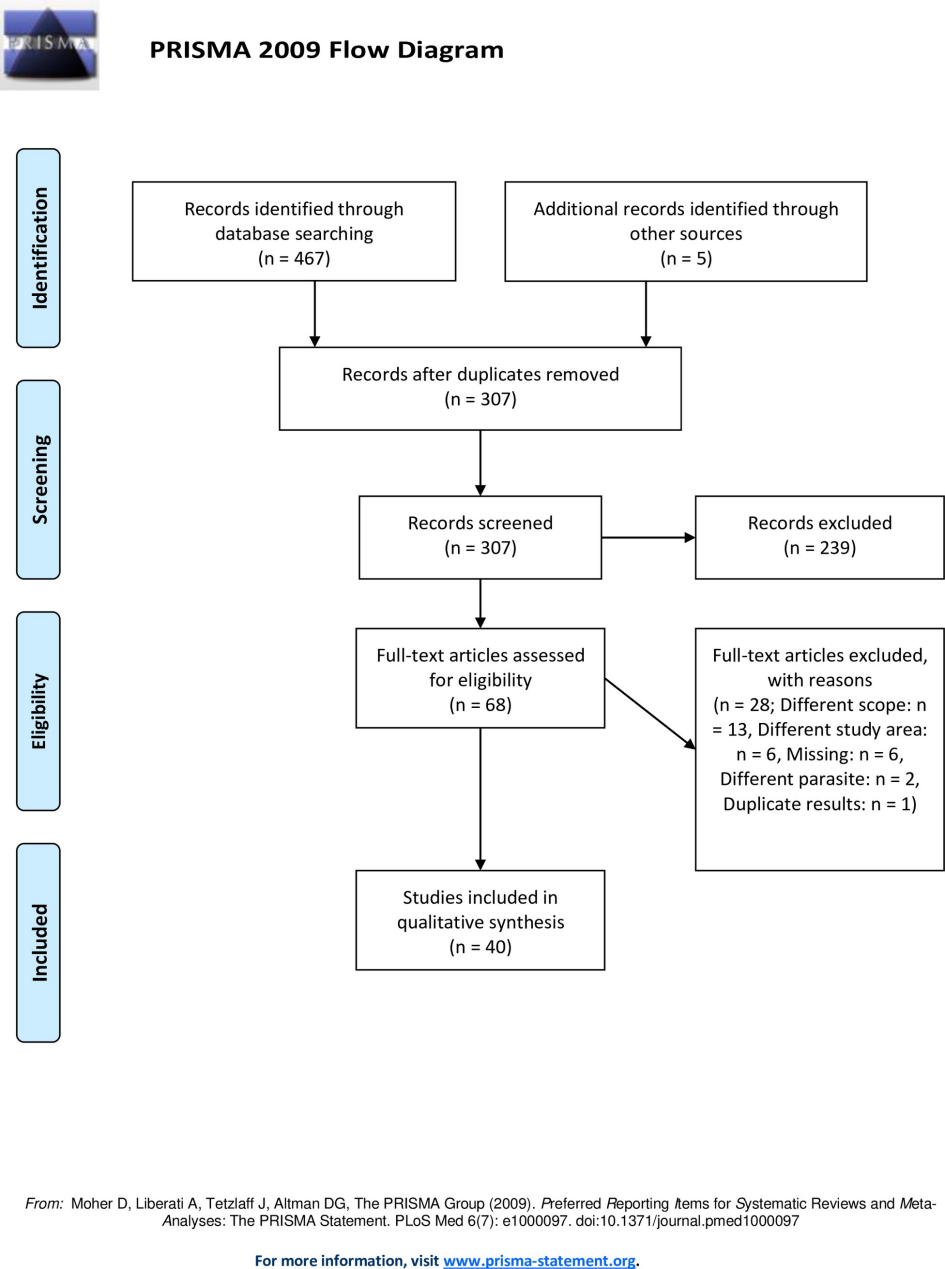
PRISMA flow diagram of a systematic review on human fascioliasis in Africa.

Datasets with retrieved records from the different databases were merged into one, after which duplicate records were removed. Then, title and abstract of retrieved records were screened for relevance. At this point, articles that focussed on non-human data, or development of diagnostic tools only as well as review articles, were removed. Subsequently, full texts of the remaining records were assessed for eligibility. Exclusion criteria were: (i) studies concerning a different parasite than *F*. *gigantica* or *F*. *hepatica*, ii) studies on fascioliasis in animals or presence of the parasite in the environment, ii) studies published before 2000 or after December 31st 2020, iii) studies reporting results from outside the study area, iv) studies reporting results out of the scope of the review question, v) duplicate records. For the third criterion, case reports for people of non-African origin diagnosed outside Africa were excluded, yet case reports for people of African origin diagnosed outside Africa area within 6 months of arrival (and with country of origin mentioned), were included. The PRISMA guidelines were followed for reporting the review [22] (Fig 1, S2 File).

### Data collection process, data items and evaluation study quality

Retrieved articles were classified as either population survey or case report, and the following variables were collected: i) population surveys: population studied, study period, population setting, number of people tested, number of positive individuals, prevalence, diagnostic tests used, risk factors associated with the disease (e.g. odds ratios), ii) case reports: study period, gender, age, clinical signs and symptoms, diagnostic tests used and test results. In case population surveys reported findings of interventions, only baseline pre-intervention data were extracted. Furthermore, publication year was extracted and it was checked whether publications were published in a journal listed with an Impact Factor (IF) in the Science Citation Index (SCI). All data were entered in preformatted tables.

Next, the study quality of population surveys was assessed by the Joanna Briggs Institute Prevalence Critical Appraisal Tool [23]. After the evaluation, the number of questions scoring a “Yes” was calculated, and divided by the total number of questions, the latter not including questions which were deemed not applicable for the study. Studies scoring less than 50%, were labelled of “weak quality”, between 50 and 75% of “moderate quality” and equal to or above 75% of “strong quality”.

### Summary measures and synthesis of results

For the population screening studies, a descriptive statistical analysis was undertaken whereby the proportion of people infected was calculated based on the number of people positive for fascioliasis and the number of people tested. The associated Wilson score 95% confidence intervals were calculated for these proportions. Chi-square tests were run to investigate the association between risk factors and presence of disease; in case of cell counts below 5, Fisher exact tests were conducted instead. Odds ratios for the risk factors were calculated as well as associated Wilson score 95% confidence intervals. The significance was set at the 5% level. All statistical analyses were carried out using R, version 3.6.1 [24].

## Results

A total of 467 publications were extracted from the three scientific databases, and 5 additional records were identified through reference list screening (Fig 1). After duplicate removal, titles and abstracts of 307 remaining records were screened for relevance. Then, the full text articles of the remaining 68 records were assessed for eligibility. Twenty-eight records were excluded at this stage: 13 were excluded due to the study topic being out of scope for the current systematic review, 6 did not focus on the study area, 2 contained data on a parasite different from *Fasciola* spp., 1 record reported duplicate results. For 6 records, the full text could not be retrieved. Finally, 40 records were retained for the qualitative synthesis.

**Fig 2:**
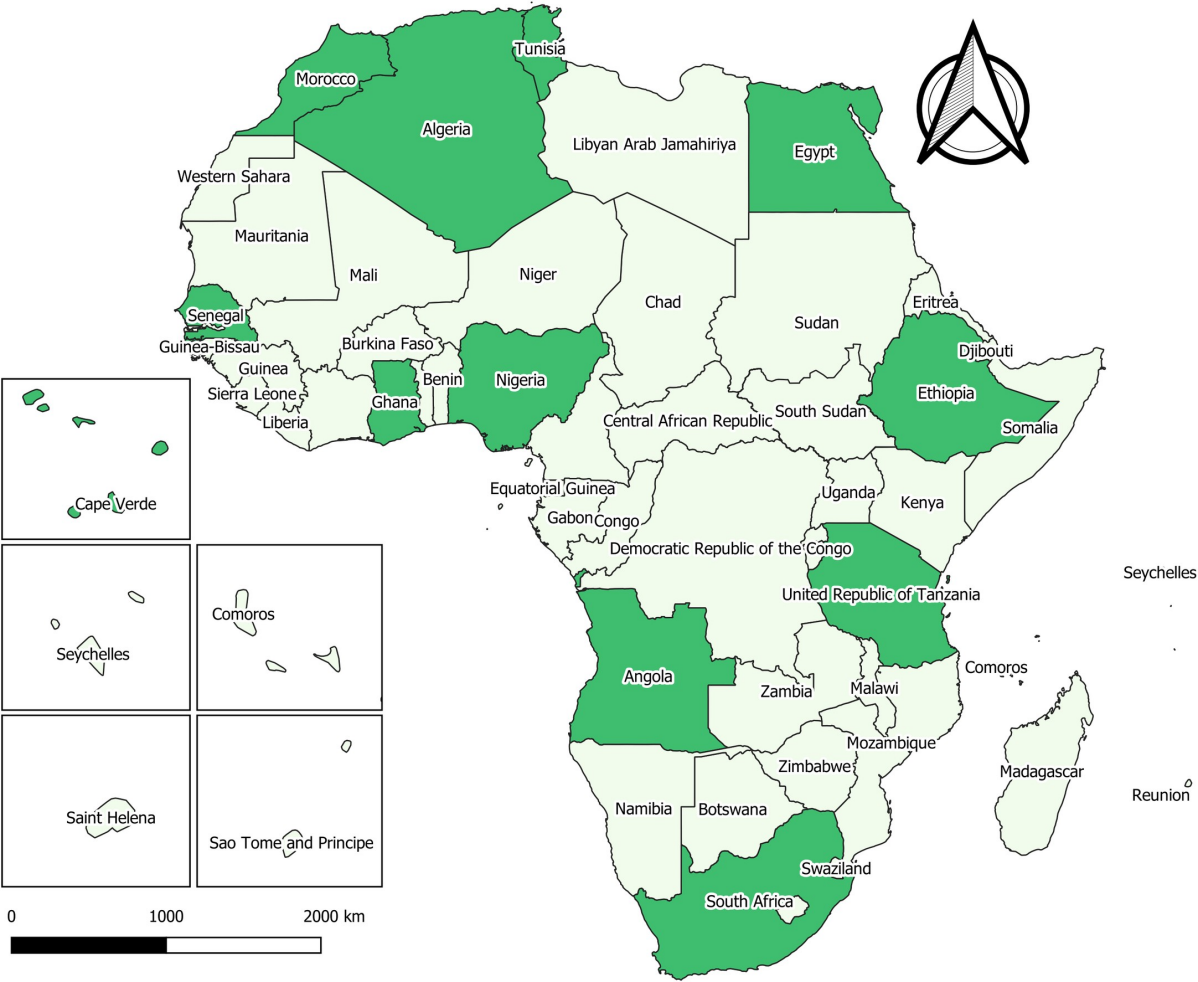
Distribution of human fascioliasis based on records retrieved in the systematic review. In dark green: presence reported, in light green: not reported or reported absent. Insert maps of islands are not presented on true scale. Shapefile republished from DIVA-GIS database (https://www.diva-gis.org/) under a CC BY license, with permission from Global Administrative Areas (GADM), original copyright 2018.

From the 40 full text articles included in the qualitative synthesis, information on human fascioliasis was retrieved for 11 African countries. The majority of studies was conducted in Egypt (n = 17), other countries included Nigeria (n = 7), Ethiopia (n = 3), South-Africa (n = 2), Tunisia (n = 3), Morocco (n = 2), Algeria (n = 1), Angola (n = 1), Cape Verde (n = 1), Ghana (n = 1), Senegal (n = 1), and Tanzania (n = 1) (Fig 2). The number of publications per year was consistently low, consistent over the study period (median: 2, minimum: 0, maximum: 5). Only twenty-two out of 40 included publications were published in a journal listed with an Impact Factor (IF) in the Science Citation Index (SCI).

A total of 28 records presented data from 36 population surveys conducted in Egypt (n = 15), Nigeria (n = 7), Angola (n = 1), Ethiopia (n = 1), Ghana (n = 1), South Africa (n = 1), Tanzania (n = 1), and Tunisia (n = 1) (Table 1), with common groups studied being the general population, schoolchildren and patients attending hospitals. The prevalence in the general population, and healthy adult and children subgroups varied widely, ranging between 0.29 and 19.3%. The most frequently used diagnostic tool was coproscopy, with only one survey providing morphometric details used for *Fasciola* spp. identification [25]. Few studies used serological methods (e.g. hemagglutination test, ELISA) [26, 27] or imaging as diagnostic tool [28]. Six studies described co-infection, mainly with *Schistosoma* spp. in surveys specifically targeting co-infections between those species [29–35]. None of the population surveys performed speciation (i.e. determination whether *Fasciola hepatica* or *F*. *gigantica* caused the infection). The population survey quality evaluation indicated that, for a good number of studies, inadequate attention was given to sample size calculation (23/28), measures to address non-responders (22/27), reliability of outcome measurement (20/28) and subject selection process (10/28). Twelve studies were scored as weak, 12 as moderate and only 4 as of strong quality (S3 File).

**Table 1:** Population surveys retrieved in a systematic review on human fascioliasis in Africa.

Country	Study Period	Study setting	Population studied	Population setting	No of people	No positive	Prevalence	95%CI	Test used	Morphometric details	Co-infections	Reference
*General population*												
Egypt	06/2000	4 villages	Villagers	Rural and Urban	678	87	12.8	10.5–15.6	C: KK	NA	Numerous cases, e.g. with *Entamoeba coli*, *E*. *histolytica/E*. *dispar*, *Endolimax nana*, *Giardia intestinalis*, *Chilomastix mesnili*, *Blastocystis hominis*, *Schistosoma*. *mansoni*, *Hymenolepis nana*	[35]
Egypt	06-09/2000	3 endemic foci (villages)	Villagers	NA	53 matched case-controls	NA	NA	NA	C: KK	NA	NA	[52]
Egypt	2003–2007	NA	Rural and Urban	NA	NA	4762	NA	NA	C: DS/FE/KK+FF	NA	NA	[53]
Egypt	04/2007-07/2007	1 village	Villagers and school-aged children	Rural	635	22	3.46	2.24–5.28	C: KK	NA	*S*. *mansoni*, *H*. *nana* (0.87%)	[34]
“	“	1 village	All villagers	NA	631	19	3.01	1.87–4.75	C: KK	NA		“
Egypt	05/2010-08/2012	4 centers	All ages	Rural and Urban	1768	11	0.62	0.33–1.15	C: DS/FE/KK	NA	NA	[27]
“	“	“	“	“	“	14	0.79	0.45–1.36	Se: IHA	NA	NA	“
“	“	“	“	“	“	20	1.13	0.71–1.77	Se: ELISA	NA	NA	“
Egypt	NA	Village	Villagers	NA	2492	200	8.03	7.00–9.18	C: KK	Histopathology	*Schistosoma* spp. (2.5%)	[33]
Egypt	NA	Village	Random sample of houses	NA	5112	382	7.47	6.77–8.24	C: KK+FF	NA	Multiple co-infection cases (71%)	[32]
Egypt	NA	One village	Random systematic sample all age groups	Urban	575	14	2.43	1.39–4.15	C: KK	NA	NA	[54]
Egypt	NA	15 villages	All villagers (> 5 years)	NA	6314	188	2.98	2.58–3.43	C: KK	NA	*Schistosoma* spp. (0.78%)	[31]
Egypt	NA	Village	Villagers	NA	1019	17	1.67	1.01–2.72	C: KK	NA	NA	[55]
Tunisia	07/2004-06/2005	Oases	Asymptomatic villagers	NA	30	2	6.67	1.16–23.5	Se: HA	NA	NA	[26]
*Healthy adult subgroup*												
Egypt	01/2005-01/2006	City	Randomly selected 20–40 years	Urban	1000	4	0.40	0.13–1.10	C: KK	NA	NA	[56]
“	“	Village	Randomly selected 20–40 years	Rural	1000	2	0.20	0.05–0.73	C: KK	NA	NA	“
Ghana	10/2014-02/2015	6 districts	Farmers	NA	95	0	0.00	0–4.84	C: FE	NA	-	[57]
Nigeria	NA	3 hospitals	Women 3rd trimester pregnancy	NA	245	0	0.00	0–1.92	C: KK	NA	-	[58]
*Healthy children subgroup*												
Angola	01/2015-05/2015	16 schools in 4 districts	Children 5–14 years	NA	230	1	0.43	0.02–2.77	C: FE	NA	NA	[59]
Egypt	1996	Households	Children 0.5–12 years entire governate	NA	1783	54	3.03	2.30–3.96	C: KK	NA	NA	[18]
“	1998	Households	Children 5–15 years from 3 districts	NA	1043	4	0.38	0.12–1.05	C: KK	NA	NA	“
“	1998	School	Children 10–12 years from 5 endemic districts	Rural	4585	171	3.73	3.21–4.33	C: KK	NA	NA	“
“	2000	School	Children 8–10 years, during parasitological monitoring	NA	1443	26	1.80	1.20–2.67	C: KK	NA	NA	“
Egypt	06-09/2000	4 endemic foci in 3 districts	Stratified sample primary schools children	NA	1331	72	5.41	4.28–6.80	C: KK	NA	NA	[60]
Ethiopia	11/2007-02/2008	6 schools	Stratified sample primary schools children	NA	520	17	3.27	1.98–5.29	C: KK	NA	Multiple cases, e.g. with *Schistosoma* spp.	[29]
Nigeria	01/1997-12/1998	20 schools	Children 2–20 years	Urban	6430	154	2.40	2.04–2.81	C: DS/FE/KK	NA	NA	[61]
Nigeria	04-06/2002	5 schools	Primary schoolchildren	NA	533	3	0.56	0.15–1.78	C: MM	NA	-	[62]
Nigeria	NA	4 schools	Children	Urban	570	5	0.88	0.32–2.16	C: FE	NA	NA	[63]
Nigeria	NA	3 communities	Children	Semi-urban	349	1	0.29	0.01–1.84	C: KK	NA	-	[58]
Nigeria	NA	2 schools (private/public)	Primary schoolchildren	Urban	254	49	19.3	14.7–24.8	C: FE	NA	NA	[64]
South Africa	04/2009-09/2009	Four schools	Primary schoolchildren	Rural and Urban	162	1	0.62	0.03–3.91	C: FE	NA	NA	[65]
*Adult patient subgroup*												
Egypt	12/2005-11/2006	University Hospital	Patients attending hospital	NA	3180	152	4.78	4.08–5.59	C: DS/FE/FT	NA	NA	[66]
Nigeria	10/2005-03/2006	Hospital	HIV-patients	NA	480	5	1.04	0.38–2.56	C: FE	NA	NA	[67]
Nigeria	NA	Hospital	Patients attending hospital	NA	438	1	0.23	0.01–1.47	C: FE	NA	NA	[68]
Tanzania	07/2012	Primary healthcare centre	Patients presenting at a primary healthcare centre	NA	1460	305	20.9	18.8–23.1	C: FE	Picture +dimensions eggs	NA	[25]
*Child patient subgroup*												
Egypt	2006–2013	Hospital	Children presenting at the hospital with focal hepatic lesions	NA	38	6	15.8	6.59–31.9	I: US/CT/MRI, Se (unspecified)	NA	NA	[28]

C: coproscopy (DS: direct smear, FE: formalin ether technique, KK: Kato-Katz thick smear, MM: McMaster, FF: Flukefinder sieving technique, FT: formalin tween), I: imaging (US: ultrasound, CT: computerized tomography, MRI: magnetic resonance imaging), Se: serology (IHA: indirect hemagglutination test), NA: not available.

Seven population surveys investigated risk factors associated with fascioliasis (Table 2) in Egypt (n = 5), Nigeria (n = 1), and Ethiopia (n = 1), all but one [27] using coproscopy for identification of positive cases. Gender (being female) and consumption of raw vegetables/seeds were identified as risk factors in 2 studies each. Other risk factors identified in the 7 studies were age, owning livestock, and use of unsafe drinking water sources.

**Table 2:** Epidemiological factors investigated in population screening studies retrieved in a systematic review on human fascioliasis in Africa.

Reference	Country	Variable	Comparison	Odds ratio (95%CI)†	p-value
[35]	Egypt	Gender	Female vs. male	1.79 (1.06–3.02)	p = 0.028
“	“	Age	6–11 vs. 1–5,	2.19 (0.94–5.07),	all p>0.050
			12–18 vs. 1–5,	1.71 (0.73–4.00),	
			>18 vs. 1–5	1.63 (0.75–3.57)	
[27]‡	Egypt	Study setting	Urban vs. rural	1.10 (0.43–2.75)	p>0.050
“	“	Gender	Female vs. male	0.87 (0.36–2.11)	p>0.050
“	“	Age	>5–20 vs. up to 5,	1.15 (0.16–12.7)§,	all p>0.05
			>20–40 vs. up to 5,	2.93 (0.62–27.7)§,	
			>40 vs. up to 5	2.04 (0.29–22.7)§	
[52]	Egypt	Eating raw seeds daily††	Yes vs. no	3.12 (1.06–9.13)	p = 0.039
“	“	Produce vegetable eaten††	Yes vs. no	2.10 (0.94–4.66)	p = 0.107
“	“	Owning cow††	Yes vs. no	2.74 (1.25–6.00)	p = 0.011
“	“	Owning buffalo††	Yes vs. no	2.52 (1.16–5.49)	p = 0.020
“	“	Owning goat††	Yes vs. no	2.40 (1.09–5.30)	p = 0.030
“	“	Bringing animals to canal for bathing/drinking††	Yes vs. no	2.35 (1.07–5.15)	p = 0.032
“	“	Owning cows and/or buffaloes††	Yes vs. no	2.35 (1.07–5.15)	p = 0.032
“	“	Owning horses and/or donkeys‡‡	Yes vs. no	2.15 (0.99–4.64)	p = 0.052
[33]	Egypt	Age	5–14 vs. below 5,	5.03 (2.54–9.95),	p<0.001,
			15–70 vs. below 5	2.19 (1.10–4.34)	p = 0.024
[54]	Egypt	Age	6–14 vs. below 6,	1.16 (0.19–12.5),	all p>0.050
			15–24 vs. below 6,	0.65 (0.05–9.12),	
			25–34 vs. below 6,	0.96 (0.07–13.6),	
			35–39 vs. below 6,	1.23 (0.09–17.4),	
			40 or older vs. below 6	0.54 (0.01–10.5)	
[29]	Ethiopia	Raw vegetable consumption	Raw vegetable consumption	8.16 (2.31–28.77) §§	p<0.001
“	“	Use of unsafe drinking water sources	Use of unsafe drinking water sources	5.91 (1.68–20.81) §§	p = 0.006
“	“	Owning sheep and/or cattle	Owning sheep and/or cattle	6.42 (1.45–28.37) §§	p = 0.014
“	“	Irrigation practices	Irrigation practices	5.93 (1.91–18.47) §§	p = 0.002
“	“	Gender	Male vs. female	2.10 (0.57–11.53)§	p>0.050
“	“	Age	per unit increase (linear)	1.14 (0.38–3.48) §§	p>0.050
[64]	Nigeria	School type	Public vs private	1.12 (0.60–2.08)	p>0.050

†Chi-square test with Wilson score 95% confidence interval, unless stated otherwise

‡All for ELISA results (as in the paper)

§Fisher exact test with 95% confidence interval

¶Only factors with p < 0.100 in article are presented here, other factors investigated can be found in Tables 3 and 4 of the reference [52]

††Reported by the mother of the household

‡‡Based on direct observation

§§As reported in the paper, no class counts available to calculate

HH = household.

Twelve case reports were retrieved for Egypt (n = 2), Ethiopia (n = 2), Morocco (n = 2), Tunisia (n = 2), Algeria (n = 1), Cape Verde (n = 1), Senegal (n = 1), and South Africa (n = 1). A total of 43 cases were described (Table 3), with an average age of 28 years old and 22 out of 43 cases being male. Fifteen cases mentioned some sort of abdominal pain, with 7 indicating that they suffered from epigastric pain specifically. Another 15 cases described fever, while 4 reported that they did not suffer from any clinical signs. For all but 3 out of 42 cases, for whom the information was available, eosinophilia was present, with percentages of eosinophils up to 80% [36]. Eight cases described eating raw wild vegetables. Out of 39 cases with coproscopy results available, *Fasciola* eggs were detected in 11. Only 5 studies provided some morphometric evidence for *Fasciola* spp. Infection, of which 2 provided pictures of eggs [37, 38], and 3 of *Fasciola* spp. Adults [39–41]. Other tests used to establish infection, were Ab-ELISA [42], electrophoresis [36, 43], hemagglutination [43] and indirect fluorescent antibody tests [44]. None of the case reports performed speciation (i.e. determination whether *Fasciola hepatica* or *F*. *gigantica* caused the infection).

**Table 3:** Case reports retrieved in a systematic review on human fascioliasis in Africa.

Country	Study Period	Gender	Age	Reported clinical symptoms	Coproscopy result	Serological test result	Morphometric evidence	Eosinophilia (Yes/no, %)	Co-infections	Anamnesis	Reference
Cape Verde	07/1998	M	67	Pain RUQ, nausea, anorexia, weight loss, diarrhea, pruritus, weakness	+	Ab-ELISA: +	NA	Yes, 35%	*Entamoeba coli*	NA	[42]
“	NA	M	33	Diffuse abdominal pain, diarrhea	-	Ab-ELISA: +	NA	Yes, 7%	*Entamoeba coli*, *Endolimax nana*	Eating watercress	“
Egypt	03/2012-12/2013	F	5	Distended abdomen	+ in 2 out of 23	NA	Picture eggs	Yes, 70%	-	Rural, farm animals	[37]
“	“	M	4	Distended abdomen				Yes, 55%	-	Rural, farm animals	“
“	“	M	10	Prolonged fever				Yes, 45%	-	Rural, farm animals	“
“	“	M	6	Prolonged fever				Yes, 70%	-	Rural, farm animals	“
“	“	F	11	Distended abdomen				Yes, 30%	-	Rural, farm animals	“
“	“	M	12	Prolonged fever				Yes, 70%	-	Rural, farm animals	“
“	“	M	14	Jaundice				Yes, 50%	-	Rural, farm animals	“
“	“	M	16	Jaundice				Yes, 55%	-	Rural, farm animals	“
“	“	M	19	None				Yes, 40%	-	Urban, no farm animals	“
“	“	F	19	Pain EG				Yes, 40%	-	Urban, no farm animals	“
“	“	M	20	Pain EG				Yes, 50%	-	Rural, farm animals	“
“	“	M	22	None				Yes, 70%	-	Rural, farm animals	“
“	“	F	22	Prolonged fever				Yes, 70%	-	Rural, farm animals	“
“	“	F	23	Prolonged fever				Yes, 30%	-	Rural, farm animals	“
“	“	M	24	Prolonged fever				Yes, 50%	-	Rural, farm animals	“
“	“	M	27	Jaundice				Yes, 60%	-	Rural, farm animals	“
“	“	F	29	Prolonged fever				Yes, 55%	-	Rural, farm animals	“
“	“	M	30	Pain EG				Yes, 30%	-	Rural, farm animals	“
“	“	M	31	Distended abdomen				Yes, 45%	-	Rural, farm animals	“
“	“	M	33	Pain EG				Yes, 30%	-	Rural, farm animals	“
“	“	F	34	Prolonged fever				Yes, 70%	-	Rural, farm animals	“
“	“	F	39	Prolonged fever				Yes, 55%	-	Urban, no farm animals	“
“	“	F	39	Prolonged fever				Yes, 35%	-	Urban, no farm animals	“
Egypt	NA	M	38	Pain EG & RUQ	NA	NA	Picture adults	Yes, 7%	NA	Farmer	[39]
Ethiopia	NA	M	65	Nausea, vomiting, fever, pain EG	+	NA	Picture egg	Yes, 16%	NA	Raw vegetable ingestion	[38]
“	NA	F	10	Anorexia, nausea, urticaria, itching, weight loss	+	NA	NA	Yes, 12%	NA	None: tap water, no raw vegetables	“
“	NA	M	70	Abdominal pain, diarrhea	+	NA	NA	Yes, 20%	NA	Rural (7 years ago), raw vegetable ingestion, drinking river water	“
“	NA	F	22	None	+	NA	Na	Yes, 10%	NA	Rural, raw vegetable ingestion, drinking river water	“
Ethiopia	NA	M	2	Chronic pain, diarrhea	+	NA	NA	Yes, 13%	NA	NA	[69]
Morocco	NA	F	40	Jaundice, fever, pain RUQ	NA	NA	Picture adult	No	NA	NA	[40]
Morocco	NA	F	6	Fever, emaciation, death	+	Ab-ELISA: +	NA	Yes, 11%	NA	Farm animals	[70]
Senegal	1993	F	41	Pain EG, colic, weight loss, dry cough	-	+	NA	Yes, 59%	-	Immigrant from Cape Verde, eating watercress, fascioliasis diagnosed in brother	[71]
“	“	M	32	Pain, colic	-	EP: +, HA: +	NA	Yes, 23%	*T*. *saginata*	Stays in Cape Verde	“
Tunisia	1999	F	46	Joint pain	-	EP: +, HA: +	NA	Yes, 52%	-	Eating wild raw plants called *telma*	[43]
Tunisia	1991	F	10	Icterus	+	NA	NA	No	NA	NA	[36]
“	1991	F	20	None	+	NA	NA	No	NA	NA	“
“	1998	F	42	Pain RUQ	NA	EP: +	NA	Yes, 50%	NA	NA	“
	2003	M	32	Weakness, weight loss, paleness	NA	EP: +	NA	Yes, 80%	NA	NA	“
Tunisia	2001	F	24	Pain RUQ	NA	NA	Picture adult	NA	NA	NA	[41]
South Africa	NA	F	73	Fever, rigor, anorexia, weight loss, cough, malaise	-	IFAT: +	NA	Yes	-	Watercress consumption	[44]
“	NA	F	37	Dyspnoea, palpitation, central chest pain, speech disorder, rash	-	IFAT: +	NA	Yes	NA	Watercress consumption, chef as job	“

Ab-ELISA: antibody-enzyme-linked immunosorbent assay, EG: epigastric, EP: electrophoresis, HA: hemagglutination, IFAT: indirect fluorescent antibody test, RUQ: right upper quadrant.

## Discussion

Human fascioliasis was found to occur in a number of countries, spread throughout the African continent. The majority of studies included in the review were conducted in Egypt, others were conducted in Algeria, Angola, Cape Verde, Ethiopia, Ghana, Morocco, Nigeria, Senegal, South-Africa, Tanzania and Tunisia. As *Fasciola* spp. are globally distributed parasites, there are no reasons to assume that the occurrence of human fascioliasis would be restricted to the countries identified in our study. As none of the studies performed speciation, no new knowledge could be retrieved about the specific distribution of human fascioliasis caused by *F*. *hepatica* and *F*. *gigantica*. It is assumed that this distribution is in line with what has been found in snails and livestock, with *F*. *gigantica* being predominant in most of the continent, except for highland areas in East Africa (Ethiopia, Tanzania, Kenya), southern Africa (Zimbabwe, Lesotho, South Africa) as well as the Mediterranean area (i.e., Maghreb countries, Egypt, Libya) where *F*. *hepatica* is the prevailing species [13, 14]. More studies on human fascioliasis should be conducted in the remaining countries in Africa, for which we could not retrieve records, and where possible, with the speciation of the causative parasite (i.e. *F*. *hepatica* or *F*. *gigantica*).

In most of the countries where population surveys were conducted, 1–2% of the screened population were found to be positive for fascioliasis. Globally, the epidemiological scenarios for fascioliasis are rather heterogeneous, ranging between zones with only imported cases, and hyperendemic areas, with prevalence estimates over 10% [45]. According to the classification proposed by Mas-Coma, Valero and Bargues [45], most of the countries for which records were retrieved would be considered meso-endemic areas, although others would rather be considered hypo- or hyperendemic. Moreover, it is well known that the prevalence of human fascioliasis does not correlate well with the prevalence of fascioliasis in livestock, the latter also generally being higher than the former [46]. This seemed also true for the prevalence of human fascioliasis in Africa, as hyperendemic areas for human fascioliasis did not always overlap with regions with high prevalence estimates in livestock, and prevalence estimates were usually lower (1–2%) than those generally reported for livestock in the region (i.e. above 10%, even above 50% at times) [15, 16]. Overall, more attention should be given to further identification of hyperendemic areas for human fascioliasis on the continent. Also, intensity of infection, measured as eggs per gram of faeces (EPG) should be reported considering that intensity is linked to pathogenicity of infection [47], and in view of the high intensities recently reported in Egypt [48].

Coproscopy was the most commonly used diagnostic tool in the retrieved population surveys. This choice might have had an impact on the prevalence estimation in the surveys. Indeed, many cases might have been missed, due to the poor sensitivity of the test, the inability to detect fascioliasis at an early stage, and the very low egg shedding, especially in low infection burdens as well as in old infections [49]. On the other hand, coproscopy can give false positive results due to spurious infection following the consumption of livers or guts, contaminated with *Fasciola* or *Paramphistomum*, and misdiagnosis of eggs from other trematodes (e.g. *Gastrodiscoides hominis*, *Paragonimus* spp.) [6]. WHO has proposed a combined evaluation of test results (e.g. serology, coproscopy) to allow differentiation of different stages of the infection, yet interpretation remains challenging [1]. Ultrasound examination might be considered another additional tool to detect fascioliasis related lesions. However, ultrasound is not always able to differentiate fascioliasis from other liver diseases because fascioliasis causes unspecific lesions and it is often unavailable in a resource-poor setting [49].

Despite the importance of the disease on the continent, the number of reports on the topic has been consistently low over the study period. The situation did not seem to improve after 2010, the year fascioliasis was listed a neglected tropical disease by the WHO [12]. Moreover, a noted discrepancy was found between the countries having reported human fascioliasis cases that were retrieved in our study as compared to the WHO fascioliasis distribution map [20], with countries present on our distribution map being absent on the WHO map (e.g. Tanzania, Angola, South Africa), and vice versa (e.g. Mali, Niger, Cameroon), although the WHO map did include information from prior 2000, in contrast to our review. This all points to limited attention for and thus knowledge on the occurrence of human fascioliasis on the African continent.

Moreover, there exists a limited understanding of the risk factors for fascioliasis in the African setting. Our search retrieved only 7 studies investigating risk factors, most of which were conducted in Egypt. In the retrieved records, consumption of raw vegetable/seed consumption and use of unsafe drinking water, both established routes of infection for fascioliasis worldwide, were significantly associated with fascioliasis [19]. Moreover, being female was found to be significantly associated with fascioliasis [35]. This could be attributed to the traditional gender roles in Africa, where women and girls are more involved in the preparation of meals, washing clothes and kitchen utensils with contaminated water than their male counterparts [30]. The background of other identified risk factors such as, owning livestock and performing irrigation practices [29] might be related to contact with contaminated water.

Apart from the risk factors identified in the studies retrieved in this systematic review, other behavioural factors might favour transmission of fascioliasis, but remain largely unstudied. For instance, authors have named traditional beverages made from sylvatic vegetables and sugarcane grown in swampy areas as source of infection in Cape Verde [19, 50]. In the Horn of Africa, chewing khat (*Catha edulis*) was linked to several fascioliasis cases in travellers [19, 50]. Other potential sources of infection might be cabbage, or other vegetables consumed uncooked that are grown in swampy areas, grass or sugar chewing [19]. Up to now, however, none of these factors have been investigated in large-scale epidemiological studies.

Our study has some limitations. First, we might have missed data, as we did not have access to potentially relevant grey literature, the amount of which might be considerable in Africa. Secondly, most retained studies, including those investigating risk factors, used coproscopy as main diagnostic test. Due to the low sensitivity and specificity of coproscopy for the diagnosis of fascioliasis [49], disease prevalence estimates might be misleading. Moreover, certain risk factors might have been missed due to the impact of the imperfect test on odds ratio estimation [51]. Nevertheless, this is the first study gathering available information on human fascioliasis epidemiology in Africa.

Overall, the low number of records retrieved indicates that human fascioliasis remains a truly neglected disease in Africa. Apart from the need for more appropriately performed screening studies to estimate its prevalence in all countries on the continent (considering its worldwide distribution), an in-depth investigation of local risk factors is lacking yet paramount to fight fascioliasis in Africa.

## Supporting information

S1 File Protocol. (DOCX)

S2 File PRISMA checklist. (DOCX)

S3 File Quality evaluation population surveys. (XLSX)

## References

[1] WHO. Report of the WHO Informal Meeting on use of triclabendazole in fascioliasis control. WHO headquarters, Geneva, Switzerland, 17–18 October 2016. Geneva; 2007.

[2] Torgerson PR. One world health: Socioeconomic burden and parasitic disease control priorities. Vet Parasitol. Elsevier B.V.; 2013;195:223–32. doi: 10.1016/j.vetpar.2013.04.004 23628712

[3] Khan MK, Sajid MS, Riaz H, Ahmad NE, He L, Shahzad M, et al. The global burden of fasciolosis in domestic animals with an outlook on the contribution of new approaches for diagnosis and control. Parasitol Res. 2013;112:2421–30. doi: 10.1007/s00436-013-3464-6 23728732

[4] Turner J, Howell A, McCann C, Caminade C, Bowers RG, Williams D, et al. A model to assess the efficacy of vaccines for control of liver fluke infection. Sci Rep. Nature Publishing Group; 2016;6:1–13. doi: 10.1038/s41598-016-0001-8 27009747 PMC4806326

[5] Chen MG, Mott KE. Progress in assessment of morbidity due to Fasciola hepatica infection: a review of recent literature. Trop Dis Bull. 1990;87:R1–R38.

[6] Mas-Coma S, Agramunt V, Valero M. Chapter 2. Neurological and Ocular Fascioliasis in Humans. Adv. Parasitol. 2014. 10.1016/B978-0-12-800099-1.00002-8 24480313

[7] Havelaar A, Kirk M, Torgerson P, Gibb H, Hald T, Lake R, et al. World Health Organization global estimates and regional comparisons of the burden of foodborne disease in 2010. PLoS Med. 2015;12:e1001923. doi: 10.1371/journal.pmed.1001923 26633896 PMC4668832

[8] Cwiklinski K, O’Neill SM, Donnelly S, Dalton JP. A prospective view of animal and human Fasciolosis. Parasite Immunol. 2016;38:558–68. doi: 10.1111/pim.12343 27314903 PMC5053257

[9] Dorny P, Praet N, Deckers N, Gabriel S. Emerging food-borne parasites. Vet Parasitol. 2009;163:196–206. doi: 10.1016/j.vetpar.2009.05.026 19559535

[10] Keiser J, Utzinger J. Emerging foodborne trematodiasis. Emerg Infect Dis. 2005;11:1507–14. doi: 10.3201/eid1110.050614 16318688 PMC3366753

[11] Mas-Coma S, Valero MA, Bargues MD. Climate change effects on trematodiases, with emphasis on zoonotic fascioliasis and schistosomiasis. Vet Parasitol. 2009;163:264–80. doi: 10.1016/j.vetpar.2009.03.024 19375233

[12] World Health Organization. Research priorities for zoonoses and marginalized infections. World Health Organ Tech Rep Ser. 2012; 23420951

[13] Esteban JG, Bargues MD, Mas-Coma S. Geographical distribution, diagnosis and treatment of human fascioliasis: a review. Res Rev Parasitol. 1998;58:13–42.

[14] Chougar L, Mas-Coma S, Artigas P, Harhoura K, Aissi M, Agramunt VH, et al. Genetically ‘pure’ Fasciola gigantica discovered in Algeria: DNA multimarker characterization, trans-Saharan introduction from a Sahel origin and spreading risk into north-western Maghreb countries. Transbound Emerg Dis. 2020;67:2190–205. doi: 10.1111/tbed.13572 32304266

[15] Mehmood K, Zhang H, Sabir AJ, Abbas RZ, Ijaz M, Durrani AZ, et al. A review on epidemiology, global prevalence and economical losses of fasciolosis in ruminants. Microb Pathog. 2017;109:253–62. doi: 10.1016/j.micpath.2017.06.006 28602837

[16] Malatji MP, Pfukenyi DM, Mukaratirwa S. Fasciola species and their vertebrate and snail intermediate hosts in East and Southern Africa: a review. J Helminthol. 2019;1–11. doi: 10.1017/S0022149X17001201 31331410

[17] Mas-Coma S. Human Fascoliasis: Epidemiological patterns in human endemic areas of South America, Africa and Asia. Southeast Asian J Trop Med Public Health. 2004;35:1–11.

[18] Curtale F, Hammoud ES, El-Wakeel A, Mas-Coma S, Savioli L. Human Fascioliasis, an emerging public health problem in the Nile Delta, Egypt. Res Rev Parasitol. 2000;60:129–34.

[19] Mas-Coma S, Bargues MD, Valero MA. Human fascioliasis infection sources, their diversity, incidence factors, analytical methods and prevention measures. Parasitology. 2018;145:1665–99. doi: 10.1017/S0031182018000914 29991363

[20] WHO. Investing to overcome the global impact of neglected tropical diseases. Third WHO report on neglected tropical diseases. Geneva; 2015.

[21] Pienaar E, Grobler L, Busgeeth K, Eisinga A, Siegfried N. Developing a geographic search filter to identify randomised controlled trials in Africa: Finding the optimal balance between sensitivity and precision. Health Info Libr J. 2011;28:210–5. doi: 10.1111/j.1471-1842.2011.00936.x 21831220

[22] Moher D, Liberati a, Tetzlaff J, Altman DG, Grp P. Preferred Reporting Items for Systematic Reviews and Meta-Analyses: The PRISMA Statement (Reprinted from Annals of Internal Medicine). Phys Ther. 2009;89:873–80. doi: 10.1371/journal.pmed.1000097 19723669

[23] Munn Z, Moola S, Riitano D, Lisy K. The development of a critical appraisal tool for use in systematic reviews addressing questions of prevalence. Int J Heal Policy Manag. 2014;3:123–8. 10.15171/ijhpm.2014.71 PMC4154549 25197676

[24] R Core Team. R: A language and environment for statistical computing. Vienna: R Foundation for Statistical Computing; 2021.

[25] Lukambagire AHS, Mchaile DN, Nyindo M. Diagnosis of human fascioliasis in Arusha region, northern Tanzania by microscopy and clinical manifestations in patients. BMC Infect Dis. BMC Infectious Diseases; 2015;15:1–8. doi: 10.1186/s12879-014-0722-x 26695775 PMC4689000

[26] Hammami H, Hamed N, Ayadi A. Epidemiological studies on Fasciola hepatica in Gafsa oases (South West of Tunisia). Parasite. 2007;14:261–4. doi: 10.1051/parasite/2007143261 17933307

[27] Adarosy H, Gad Y, El-Baz S, El-Shazly A. Changing pattern of fascioliasis prevalence early in the 3rd milennium in Dakahlia Governate, Egypt: un update. J Egypt Soc Parasitol. 2013;43:275–86. doi: 10.12816/0006384 23697033

[28] El-Karaksy H, Mogahed E, El-Sayed R, El-Raziky M, Sheba M, Besheer M, et al. Focal hepatic lesions in Egyptian infants and children: the pediatric hepatologist perspective. Minerva Pediatr. 2018;70:35–45. doi: 10.23736/S0026-4946.17.04299-2 25926159

[29] Fentie T, Erqou S, Gedefaw M, Desta A. Epidemiology of human fascioliasis and intestinal parasitosis among schoolchildren in Lake Tana Basin, northwest Ethiopia. Trans R Soc Trop Med Hyg. 2013;107:480–6. doi: 10.1093/trstmh/trt056 23843557

[30] Curtale F, Hassanein YAW, Barduagni P, Yousef MM, El-Wakeel A, Hallaj Z, et al. Human fascioliasis infection: gender differences within school-age children from endemic areas of the Nile Delta, Egypt. Trans R Soc Trop Med Hyg. 2007;101:155–60. doi: 10.1016/j.trstmh.2006.05.006 16890257

[31] Osman MM, Shehab AY, Zaki A, Farag HF. Evaluation of two doses of triclabendazole in treatment of patients with combined schistosomiasis and fascioliasis. East Mediterr Heal J. 2011;17:266–70. 10.26719/2011.17.4.266 22259882

[32] El-Shazly A, Soliman M, Gabr A, Haseeb A, Morsy A, Arafa M, et al. Clinico-epidemiological study of human fascioliasis in an endemic focus in Dakahlia Governate, Egypt. J Egypt Soc Parasitol. 2001;31:725–36. 11775099

[33] Abou-Basha L, Salem A, Osman M, El-Hefni S, Zaki A. Hepatic fibrosis due to fascioliasis and/or schistosomiasis in Abis 1 village, Egypt. La Rev Santé la Méditerranée Orient. 2000;3:870–8. 12197343

[34] Keiser J, Sayed H, El-Ghanam M, Sabry H, Anani S, El-Wakeel A, et al. Efficacy and safety of artemether in the treatment of chronic fascioliasis in Egypt: Exploratory phase-2 trials. PLoS Negl Trop Dis. 2011;5:e1285. doi: 10.1371/journal.pntd.0001285 21909440 PMC3167773

[35] Esteban J-G, Gonzalez C, Curtale F, Muñoz-Antoli C, Valero M, Bargues M, et al. Hyperendemic fascioliasis associated with schistosomiasis in villages in the Nile Delta of Egypt. Am J Trop Med Hyg. 2003;69:429–37. 14640504

[36] Zait H, Hamrioui B. Nouveaux cas de fasciolose humaine en Algérie. Médecine Trop. 2005;65:395–6. 16548499

[37] Mekky MA, Tolba M, Abdel-Malek MO, Abbas WA, Zidan M. Human fascioliasis: A re-emerging disease in Upper Egypt. Am J Trop Med Hyg. 2015;93:76–9. doi: 10.4269/ajtmh.15-0030 25870421 PMC4497909

[38] Bayu B, Asnake S, Woretaw A, Ali J, Gedefaw M, Fente T, et al. Cases of human fascioliasis in North-West Ethiopia. Ethiop J Heal Dev. 2011;19:237–40. 10.4314/ejhd.v19i3.10004

[39] Makhlouf NA, Moustafa E, Zakaria M, Imam HM. Fascioliasis: A report on a case presenting with abdominal pain. Arab J Gastroenterol. Pan-Arab Association of Gastroenterology; 2017;18:172–3. doi: 10.1016/j.ajg.2017.05.018 28988787

[40] Lefryekh R, Bensaad A, Bensardi F, Elhattabi K, Bouali M, Daif B, et al. Hepatic fascioliasis presenting with bile duct obstruction: a case report. Pan Afr Med J. 2017;28:44. doi: 10.11604/pamj.2017.28.44.11532 29158867 PMC5687870

[41] Khelifi S, Bouhafa A, Ouertani F, Maamer A, Hedfi M, Cherif A, et al. Distomatose de la voie biliaire principale traitée par voie coelioscopique. A propos d’un cas. Tunis Med. 2006;84:385–6. 17042216

[42] Graham CS, Brodie SB, Weller PF. Imported Fasciola hepatica infection in the United States and treatment with triclabendazole. Clin Infect Dis. 2002;33:1–5. 10.1086/320870 11389487

[43] Sellami H, Elloumi M, Cheikhrouhou F, Makni F, Baklouti S, Ayadi A. Fasciola hepatica infestation with joint symptoms. Jt Bone Spine. 2003;70:71–2. doi: 10.1016/s1297-319x(02)00017-9 12639623

[44] Black J, Ntusi N, Stead P, Mayosi B, Mendelson M. Human fascioliasis in South Africa. South African Med J. 2013;103:658–9. doi: 10.7196/samj.7184 24300687

[45] Mas-Coma S, Valero M, Bargues M. Chapter 2 Fasciola, Lymnaeids and Human Fascioliasis, with a Global Overview on Disease Transmission, Epidemiology, Evolutionary Genetics, Molecular Epidemiology and Control. 1st ed. Adv. Parasitol. Elsevier Ltd.; 2009. 10.1016/S0065-308X(09)69002-3 19622408

[46] Mas-Coma S, Bargues MD, Valero MA. Fascioliasis and other plant-borne trematode zoonoses. Int J Parasitol. 2005;35:1255–78. doi: 10.1016/j.ijpara.2005.07.010 16150452

[47] Montresor A, Crompton DWT, Bundy DAP, Hall A, Savioli L. Guidelines for the Evaluation of Soil-Transmitted Helminthiasis and Schistosomiasis at Community Level. A Guide for Managers of Control Programmes; WHO/CTD/SIP/98.1. Geneva, Switzerland; 1998.

[48] Periago MV, Valero MA, Artigas P, Agramunt H, Bargues MD, Curtale F, et al. Very high fascioliasis intensities in schoolchildren from Nile Delta Governorates, Egypt: The Old World highest burdens found in lowlands. Pathogens. 2021;10:1210. doi: 10.3390/pathogens10091210 34578242 PMC8470878

[49] Mas-Coma S, Bargues M, Valero MA. Diagnosis of human fascioliasis by stool and blood techniques: update for the present global scenario. Parasitology. 2014;141:1918–46. doi: 10.1017/S0031182014000869 25077569

[50] Ashrafi K, Bargues MD, O’Neill S, Mas-Coma S. Fascioliasis: A worldwide parasitic disease of importance in travel medicine. Travel Med Infect Dis. Elsevier Ltd; 2014;12:636–49. doi: 10.1016/j.tmaid.2014.09.006 25287722

[51] Valle D, Lima JMT, Millar J, Amratia P, Haque U. Bias in logistic regression due to imperfect diagnostic test results and practical correction approaches. Malar J. BioMed Central; 2015;14:1–9. doi: 10.1186/s12936-015-0966-y 26537373 PMC4634725

[52] Curtale F, Mas-Coma S, Hassanein YAW, Barduagni P, Pezzotti P, Savioli L. Clinical signs and household characteristics associated with human fascioliasis among rural population in Egypt: A case-control study. Parassitologia. 2003;45:5–11. 15270537

[53] El-Shazly AM, El-Beshbishi SN, Azab MS, El-Malky M, Abdeltawab AH, Morsy ATA. Past and present situation of human fascioliasis in Dakahlia Governorate, Egypt. J Egypt Soc Parasitol. 2009;39:247–62. 19530625

[54] Fawzi M, El-Sahn AA, Ibrahim HF, Shehata AI. Vegetable-transmitted parasites among inhabitants of El-Prince, Alexandria and its relation to housewives’ knowledge and practices. J Egypt Public Health Assoc. 2004;79:13–29. 16916047

[55] Abo-Madyan AA, Morsy TA, Motawea SM, Morsy ATA. Clinical trial of Mirazid in treatment of human fascioliasis, Ezbet El-Bakly (Tamyia Center) Al-Fayoum Governorate. J Egypt Soc Parasitol. 2004;34:807–18. 15587309

[56] El-Shazly A, El-Nahas H, Soliman M, Sultan D, Abedl Tawab A, Morsy T. The reflection of control programmes of parasitic diseases. J Egypt Soc Parasitol. 2006;36:467–80. 16927862

[57] Squire SA, Yang R, Robertson I, Ayi I, Squire DS, Ryan U. Gastrointestinal helminths in farmers and their ruminant livestock from the Coastal Savannah zone of Ghana. Parasitol Res. Parasitology Research; 2018;117:3183–94. doi: 10.1007/s00436-018-6017-1 30030626

[58] Arinola GO, Morenikeji OA, Akinwande KS, Alade AO, Olateru-Olagbegi O, Alabi PE, et al. Serum Micronutrients in helminth-infected pregnant women and children: Suggestions for differential supplementation during anti-helminthic treatment. Ann Glob Heal. Elsevier Inc; 2015;81:705–10. doi: 10.1016/j.aogh.2015.10.001 27036729

[59] De Alegría MLAR, Colmenares K, Espasa M, Amor A, Lopez I, Nindia A, et al. Prevalence of Strongyloides stercoralis and other intestinal parasite infections in school children in a rural area of Angola: a cross-sectional study. Am J Trop Med Hyg. 2017;97:1226–31. doi: 10.4269/ajtmh.17-0159 28820707 PMC5637607

[60] Curtale F, El-Wahab Hassanein YA, El Wakeel A, Mas-Coma S, Montresor A. Distribution of human fascioliasis by age and gender among rural population in the Nile Delta, Egypt. J Trop Pediatr. 2003;49:264–8. doi: 10.1093/tropej/49.5.264 14604157

[61] Okaka C, Awharitoma A, Okonji J. Gastrointestinal parasites of school children in Benin city, Nigeria. Iran J Public Health. 2000;29:1–12.

[62] Ijagbone I, Olagunju T. Intestinal helminth parasites in school children in Iragbiji, Boripe Local Government, Osun State, Nigeria. African J Biomed Res. 2006;9:63–6.

[63] Ihesiulor G, Kashibu E, Azeez-Akande O, Imoru M. Helminths of the gastrointestinal tract among children in Kano, Northern Nigeria. Asian J Biol Life Sci. 2013;2:122–6.

[64] Shitta K, Audu H, Usman A. Prevalence of geohelminthes in school children in some parts of Lokoja, Kogi State, North-Central Nigeria. Bayero J Pure Appl Sci. 2017;10:151–4.

[65] Nxasana N, Baba K, Bhat V, Vasaikar S. Prevalence of intestinal parasites in primary school children of Mthatha, Eastern Cape Province, South Africa. Ann Med Health Sci Res. 2013;3:511–6. doi: 10.4103/2141-9248.122064 24380000 PMC3868115

[66] El-Shazly A, Awad S, Sultan D, Sadek G, Khalil H, Morsy T. Intestinal parasites in Dakahlia Governate, with different techniques in diagnosing protozoa. J Egypt Soc Parasitol. 2006;36:1023–34. 17153711

[67] Abaver DT, Nwobegahay JM, Goon DT, Iweriebor BC, Khoza LB. Enteric parasitic infections in HIV-infected patients with low CD4 counts in Toto, Nigeria. Pakistan J Med Sci. 2012;28:630–3. 10.12669/pjms.284.2296

[68] Na’acha E, Vandi P, Chessed G. Species and prevalence determination of Human Intestinal Parasites among Patients attending two Medical Centers in Yola, Adamawa State, Nigeria. J Appl Sci Environ Manag. 2017;21:431. 10.4314/jasem.v21i3.4

[69] Jensenius M, Flægstad T, Stenstad T, Gjølberg T, Vlatkovic L, Schjøth-Iversen L, et al. Fascioliasis imported to Norway. Scand J Infect Dis. 2005;37:534–7. doi: 10.1080/00365540510034518 16012024

[70] Kelgeri C, Valamparampil J, Shanmugam N, Srinivas Reddy M, Swaminathan S, Rela M. An unusual cause of graft loss in pediatric liver transplant recipient—Fasciola hepatica. Pediatr Transplant. 2019;23:3–5. doi: 10.1111/petr.13521 31240781

[71] Ka M, Mbengue M, Diop B, Pouye A, Da Veiga J, Dia D, et al. Two unexpected cases of hepatobiliary fascioliasis in Dakar. Dakar Médical. 2002;47:202–5. 15776676

