# Supplementary material for: Human fascioliasis in Africa: A systematic review
Source: PLoS One. 2021 Dec 9;16(12):e0261166. doi: 10.1371/journal.pone.0261166 (PMC8659297; doi:10.1371/journal.pone.0261166)
Supplement: S1 File — (DOCX) [file pone.0261166.s001.docx]

Human fascioliasis in Africa: a systematic review: Protocol

**Aim:** The aim was to summarize current knowledge of human fasicoliasis in Africa.

**Research questions:**

-What is the distribution of human fascioliasis in Africa?

-What is reported prevalence range of human fascioliasis in Africa?

-What are risk factors reported for human fascioliasis in Africa?

**Methods:** The systematic review followed the PRISMA (Preferred Reporting Items for Systematic Reviews and Meta-Analyses) guidelines for reporting systematic reviews (Moher et al. 2009). In brief, after extracting records from the different sources, and removal of duplicates, title/abstracts were screened for relevance. Then, full text articles of retained records were evaluated for eligibility and data were extracted for included records.

**Sources:**

-Bibliographic databases: Pubmed (<https://pubmed.ncbi.nlm.nih.gov/>), Web of Science (<https://webofknowledge.com>), Africa Wide Information (<https://cabdirect.org>).

-Additional sources: reference lists of retained records and/or review articles were screened for additional sources.

**Search phrase:**

(human OR woman OR man OR girl OR boy OR patient OR patients OR child OR children OR zoonotic OR zoonos*) AND (“F. hepatica” OR “F. gigantica” OR fasciol*) AND ((Africa OR African continent OR Africa OR Algeria OR Angola OR Benin OR Botswana OR Burkina Faso OR Burundi OR Cameroon OR Canary Islands OR Cape Verde OR Central African Republic OR Chad OR Comoros OR Congo OR Democratic Republic of Congo OR Djibouti OR Egypt OR Equatorial Guinea OR Eritrea OR Ethiopia OR Gabon OR Gambia OR Ghana OR Guinea OR Guinea Bissau OR Ivory Coast OR Cote d’Ivoire OR Jamahiriya OR Jamahiryia OR Kenya OR Lesotho OR Liberia OR Libya OR Libia OR Madagascar OR Malawi OR Mali OR Mauritania OR Mauritius OR Mayote OR Morocco OR Mozambique OR Mocambique OR Namibia OR Niger OR Nigeria OR Principe OR Reunion OR Rwanda OR Sao Tome OR Senegal OR Seychelles OR Sierra Leone OR Somalia OR South Africa OR St Helena OR Sudan OR Swaziland OR Tanzania OR Togo OR Tunisia OR Uganda OR Western Sahara OR Zaire OR Zambia OR Zimbabwe OR Central Africa OR Central African OR West Africa OR West African OR Western Africa OR Western African OR East Africa OR East African OR Eastern Africa OR Eastern African OR North Africa OR North African OR Northern Africa OR Northern African OR South African OR Southern Africa OR Southern African OR sub Saharan Africa OR sub Saharan African OR subSaharan Africa OR subSaharan African) NOT (guinea pig OR guinea pigs OR aspergillus niger))

*Note: We based the part of the search phrase describing the African continent on the search phrase developed by Pienaar et al. (2011)*

**Search phrase translated for use in PubMed:** (human OR woman OR man OR girl OR boy OR patient OR patients OR child OR children OR zoonotic OR zoonos*) AND (“F. hepatica” OR “F. gigantica” OR fasciol*) AND ((Africa OR "African continent" OR “Africa”[MeSH] OR Africa*[tw] OR Algeria[tw] OR Angola[tw] OR Benin[tw] OR Botswana[tw] OR “Burkina Faso”[tw] OR Burundi[tw] OR Cameroon[tw] OR “Canary Islands”[tw] OR “Cape Verde”[tw] OR “Central African Republic”[tw] OR Chad[tw] OR Comoros[tw] OR Congo[tw] OR “Democratic Republic of Congo”[tw] OR Djibouti[tw] OR Egypt[tw] OR “Equatorial Guinea”[tw] OR Eritrea[tw] OR Ethiopia[tw] OR Gabon[tw] OR Gambia[tw] OR Ghana[tw] OR Guinea[tw] OR “Guinea Bissau”[tw] OR “Ivory Coast”[tw] OR “Cote d’Ivoire”[tw] OR Jamahiriya[tw] OR Jamahiryia[tw] OR Kenya[tw] OR Lesotho[tw] OR Liberia[tw] OR Libya[tw] OR Libia[tw] OR Madagascar[tw] OR Malawi[tw] OR Mali[tw] OR Mauritania[tw] OR Mauritius[tw] OR Mayote[tw] OR Morocco[tw] OR Mozambique[tw] OR Mocambique[tw] OR Namibia[tw] OR Niger[tw] OR Nigeria[tw] OR Principe[tw] OR Reunion[tw] OR Rwanda[tw] OR “Sao Tome”[tw] OR Senegal[tw] OR Seychelles[tw] OR “Sierra Leone”[tw] OR Somalia[tw] OR “South Africa”[tw] OR “St Helena”[tw] OR Sudan[tw] OR Swaziland[tw] OR Tanzania[tw] OR Togo[tw] OR Tunisia[tw] OR Uganda[tw] OR “Western Sahara”[tw] OR Zaire[tw] OR Zambia[tw] OR Zimbabwe[tw] OR “Central Africa”[tw] OR “Central African”[tw] OR “West Africa”[tw] OR “West African”[tw] OR “Western Africa”[tw] OR “Western African”[tw] OR “East Africa”[tw] OR “East African”[tw] OR “Eastern Africa”[tw] OR “Eastern African”[tw] OR “North Africa”[tw] OR “North African”[tw] OR “Northern Africa”[tw] OR “Northern African”[tw] OR “South African”[tw] OR “Southern Africa”[tw] OR “Southern African”[tw] OR “sub Saharan Africa”[tw] OR “sub Saharan African”[tw] OR “subSaharan Africa”[tw] OR “subSaharan African”[tw]) NOT (“guinea pig”[tw] OR “guinea pigs”[tw] OR “aspergillus niger”[tw]))

**Search phrase translated for use in Web of Science and CAB Direct:** (human OR woman OR man OR girl OR boy OR patient OR patients OR child OR children OR zoonotic OR zoonos*) AND (“F. hepatica” OR “F. gigantica” OR fasciol*) AND ((Africa OR “African continent” OR Africa* OR Algeria OR Angola OR Benin OR Botswana OR “Burkina Faso” OR Burundi OR Cameroon OR “Canary Islands” OR “Cape Verde” OR “Central African Republic” OR Chad OR Comoros OR Congo OR “Democratic Republic of Congo” OR Djibouti OR Egypt OR “Equatorial Guinea” OR Eritrea OR Ethiopia OR Gabon OR Gambia OR Ghana OR Guinea OR “Guinea Bissau” OR “Ivory Coast” OR “Cote d’Ivoire” OR Jamahiriya OR Jamahiryia OR Kenya OR Lesotho OR Liberia OR Libya OR Libia OR Madagascar OR Malawi OR Mali OR Mauritania OR Mauritius OR Mayote OR Morocco OR Mozambique OR Mocambique OR Namibia OR Niger OR Nigeria OR Principe OR Reunion OR Rwanda OR Sao Tome OR Senegal OR Seychelles OR “Sierra Leone” OR Somalia OR South Africa OR St Helena OR Sudan OR Swaziland OR Tanzania OR Togo OR Tunisia OR Uganda OR “Western Sahara” OR Zaire OR Zambia OR Zimbabwe OR “Central Africa” OR “Central African” OR “West Africa” OR “West African” OR “Western Africa” OR “Western African” OR “East Africa” OR “East African” OR “Eastern Africa” OR “Eastern African” OR “North Africa” OR “North African” OR “Northern Africa” OR “Northern African” OR “South African” OR “Southern Africa” OR “Southern African” OR “sub Saharan Africa” OR “sub Saharan African” OR “subSaharan Africa” OR “subSaharan African”) NOT (“guinea pig” OR “guinea pigs” OR “aspergillus niger”))

**Inclusion/exclusion criteria:**

- **Exclusion criteria**
- Studies concerning a different parasite than *F. gigantica* or *F. hepatica.*
- Studies on fascioliasis in animals.
- Studies published before 2000 or after December 31^st^ 2020
- Studies reporting results from outside the study area (unless case reports for people of African origin diagnosed outside Africa area within 6 months of travel (and with country of origin mentioned))
- Studies reporting results out of the scope of the review question.
- Duplicate records.
- **Inclusion criteria**
- Studies reporting data on human fascioliasis from the African continent.

**Variables extracted:** For population surveys, the following variables were extracted: population studied, study period, population setting, number of people tested, number of positive individuals, prevalence, diagnostic tests used, risk factors associated with the disease (e.g. odds ratios). Only baseline pre-intervention data were extracted from population surveys reporting interventions. For case reports, the following variables were extracted: study period, gender, age, clinical signs and symptoms, diagnostic tests used and test results. Furthermore, author, reference and publication year were extracted from each retained record.

**New data added:** For each of the retained records, the Impact Factor (IF) in the Science Citation Index (SCI) of the journal was retrieved. Population surveys were also evaluated using the Joanna Briggs Institute Prevalence Critical Appraisal Tool (Munn et al. 2014).

**Languages:** English articles.

**Study period:** 1^st^ January 2000 – 31^st^ December 2020

**Geographical range:** All countries within the African continent.

**References:**

*Moher D, Liberati A, Tetzlaff J, Altman DG, The PRISMA Group (2009). Preferred Reporting Items for Systematic Reviews and Meta-Analyses: The PRISMA Statement (Reprinted from Annals of Internal Medicine). Physical Therapy, 89(7):873–880.*

*Munn, Z., Moola, S., Riitano, D., & Lisy, K. (2014). The development of a critical appraisal tool for use in systematic reviews addressing questions of prevalence. International Journal of Health Policy and Management, 3(3), 123–128. https://doi.org/10.15171/ijhpm.2014.71*

*Pienaar E, Grobler L, Busgeeth K, Eisinga A, Siegfried N (2011). Developing a geographic search filter to identify randomised controlled trials in Africa: finding the optimal balance between sensitivity and precision. Health Information and Libraries Journal, 28(3):210-5.*
